# Human first-trimester chorionic villi have a myogenic potential

**DOI:** 10.1007/s00441-012-1340-9

**Published:** 2012-02-28

**Authors:** Reiko Arakawa, Ryoko Aoki, Masayuki Arakawa, Kayoko Saito

**Affiliations:** 1 Affiliated Field of Medical Genetics, Division of Biomedical Engineering and Science, Graduate Course of Medicine, Graduate School of Tokyo Women’s Medical University, 10-22 Kawadacho, Shinjyuku, Tokyo, 162-0054 Japan; 2 Institute of Medical Genetics, Tokyo Women’s Medical University, 10-22 Kawadacho, Shinjyuku, Tokyo, 162-0054 Japan; 3 Institute of Microbial Chemistry, Microbial Chemistry Research Foundation, 3-14-23 Kamiosaki, Shinagawa, Tokyo, 141-0021 Japan

**Keywords:** Duchenne muscular dystrophy, First-trimester chorionic villi, Myogenic differentiation, Dystrophin, Cell therapy

## Abstract

First-trimester chorionic-villi-derived cells (FTCVs) are the earliest fetal material that can be obtained for prenatal diagnosis of fetal disorders such as Duchenne muscular dystrophy (DMD). DMD is a devastating X-linked disorder characterized by the absence of dystrophin at the sarcolemma of muscle fibers. Currently, a limited number of treatment options are available for DMD, although cell therapy is a promising treatment strategy for muscle degeneration in DMD patients. A novel candidate source of cells for this approach is FTCVs taken between the 9th and 11th weeks of gestation. FTCVs might have a higher undifferentiated potential than any other tissue-derived cells because they are the earliest fetal material. We examined the expression of mesenchymal stem cell and pluripotent stem cell markers in FTCVs, in addition to their myogenic potential. FTCVs expressed mesenchymal stem cell markers and *Nanog* and *Sox2* transcription factors as pluripotent stem cell markers. These cells efficiently differentiated into myotubes after myogenic induction, at which point *Nanog* and *Sox2* were down-regulated, whereas *MyoD*, *myogenin*, *desmin* and *dystrophin* were up-regulated. To our knowledge, this is the first demonstration that FTCVs can be efficiently directed to differentiate in vitro into skeletal muscle cells that express dystrophin as the last stage marker of myogenic differentiation. The myogenic potential of FTCVs reveals their promise for use in cell therapy for DMD, for which no effective treatment presently exists.

**Electronic supplementary material:**

The online version of this article (doi:10.1007/s00441-012-1340-9) contains supplementary material, which is available to authorized users.

## Introduction

In the early 1980s, prenatal diagnosis by using chorionic villus sampling (CVS) was introduced; this method has advanced the diagnosis of fetal genetic disorders such as Duchenne muscular dystrophy (DMD) in the first-trimester of pregnancy. The numerous advances in prenatal diagnosis and molecular genetic testing of DMD have permitted the accurate and early diagnosis of carrier women.

During human development, the morula constitutes the inner cell mass and the outer cell mass. The outer cell mass forms the trophoblasts, which later contribute to the formation of the placenta. In the early weeks of development, villi derived from the trophoblasts give rise to the chorionic villi (Sadler 2009). The first-trimester chorionic villi can be collected for CVS. By the third trimester of pregnancy, the placenta consists in two components: a fetal portion, which is derived from the chorionic villus, the smooth chorion, the chorionic plate and the amnion and a maternal portion.

Of note, the first-trimester chorionic-villi-derived cells (FTCVs) are the earliest fetal material that can be obtained during pregnancy. Despite the presence of stem cells in the FTCV population, only a few detailed reports have been presented on the differentiation capability of fetal cells obtained from CVS (Poloni et al. 2008; Spitalieri et al. 2009). Furthermore, third-trimester placental chorionic-villi-derived cells (TTCVs) are either myogenic progenitors or have myogenic potential (Kawamichi et al. 2010).

DMD is the most common and severe form of muscular dystrophy. DMD is an X-linked recessive genetic disease that affects 1 in 3,500 newborn male children and is caused by a deficiency in dystrophin, which is associated with a large oligomeric complex of glycoproteins that link the cytoskeleton to the extracellular matrix (Ervasti and Campbell 1991). The absence of dystrophin results in the destabilization of the architecture of the extracellular membrane/sarcolemma cytoskeleton, making muscle fibers susceptible to contraction-associated mechanical stress and degeneration.

Unfortunately, no curative therapy is available for DMD, although a few novel treatments, including pharmacologic agents and genetic alterations for replacing the missing dystrophin by exon skipping or viral gene delivery, are in clinical trials (Kinali et al. 2009; Miyagoe-Suzuki and Takeda 2010). Another attractive alternative for treating DMD is cell therapy for every gene mutation in patients. In particular, myoblasts are the first choice as cellular therapeutics for skeletal muscle, because of their intrinsic myogenic commitment (Grounds et al. 2002). However, myoblasts recovered from muscle biopsies are known to be poorly expandable in vitro and rapidly senesce (Cossu and Mavilio 2000). Therefore, an alternative source of muscle progenitor cells is desirable. Accordingly, researchers have become interested in a variety of other cells with myogenic potential, including bone marrow stromal cells (Saburina et al. 2004), blood-vessel-associated mesoangioblasts (Sampaolesi et al. 2006), umbilical cord blood cells (Gang et al. 2004), adipose tissue cells (Di Rocco et al. 2006), endometrial and menstrual blood cells (Cui et al. 2007), placenta cells (Kawamichi et al. 2010) and induced pluripotent stem cells from DMD (Kazuki et al. 2010).

In this study, we have identified a subpopulation of cells that express various markers of mesenchymal stem cells (MSCs) and pluripotent stem cells in the larger FTCV population. Subsequently, we have found that directed differentiation of the FTCVs into skeletal muscle cells in vitro efficiently generates dystrophin-positive myotubes.

## Materials and methods

### Isolation of first-trimester chorionic villi and placental tissues

First-trimester chorionic villi were excised from subjects undergoing prenatal diagnosis between the 9th and 11th weeks of gestation. Each biopsy was performed by using standard procedures under continuous ultrasound guidance. The human placentas were collected between the 37th and 41st weeks of gestation (third trimester) during normal full-term deliveries. For this study, the first-trimester chorionic villi were obtained from 12 individuals and placentas were obtained from five individuals. Of note, the cells from both these sources are established as having the normal full-length *dystrophin* gene without any mutations. Informed patient consent was obtained in all cases. Ethical approval for tissue collection was granted by the Institutional Review Board of Tokyo Women’s Medical University, Japan.

### Tissue and cell culture

All the first-trimester chorionic villi and placentas were processed within 24 h of collection. After the villi had been cut into small pieces, the tissues were cultured in AmnioMAX-C100 medium (Invitrogen, Carlsbad, Calif., USA), whereas the placentas were washed extensively with phosphate-buffered saline (PBS). Once the amnion had been peeled off, each placenta was separated into three parts, namely, the chorionic villus, chorionic plate and decidua basalis. To isolate TTCVs, placental chorionic villi were subsequently minced by using scissors and re-suspended in MSC basal medium (Lonza, Walkersville, Md., USA). The FTCVs and TTCVs were maintained separately at 37°C in a humidified atmosphere containing 5% CO_2_ and were allowed to attach to their culture vessels. After cells had attached to their vessels, the medium was regularly changed twice a week. Once the cells had grown to 70%–80% confluence, they were harvested with trypsin (0.25%) and 1 mM EDTA (0.02%) in PBS (1:1 v/v) and were subsequently plated onto new dishes. After the second passage, FTCVs were cultured in MSC basal medium.

The pluripotent human embryonal carcinoma cell line NTERA-2 clone D1 was purchased from the European Collection of Cell Cultures (Porton Down, Salisbury, UK). Normal human dermal fibroblasts (HDFs) were purchased from Lonza. NTERA-2 and HDFs were cultured in Dulbecco’s modified Eagle’s medium (DMEM) supplemented with 10% fetal bovine serum (FBS) and 2 mM glutamine.

### Flow cytometric analysis

FTCVs and TTCVs obtained between the third and sixth passages of culture were characterized by flow cytometric analysis. Antibodies against human CD29, CD34, CD44, CD73 and CD105 were purchased from Beckman Coulter (Brea, Calif., USA) and BD Biosciences Pharmingen (San Diego, Calif., USA). Briefly, the cells were stained with primary antibodies and fluorescently labeled secondary antibodies, each for 20 min at 4°C. The cells were then assessed on an EPICS XL-MCL analyzer (Beckman Coulter). Three independent experiments were performed in triplicate.

### Quantitative analysis by real-time and semi-quantitative reverse transcription with polymerase chain reaction

Total RNA was prepared by using TRIzol Reagent (Invitrogen). Human skeletal muscle total RNA was purchased from Clontech Laboratories (Mountain View, Calif., USA). An aliquot of 0.1–2 μg total RNA was reverse-transcribed into cDNA with SuperScript III Reverse Transcriptase (Invitrogen) according to the manufacturer’s protocol.

For quantitative real-time reverse transcription (RT) with polymerase chain reaction (PCR) analysis (qPCR), PCR was performed by using a Fast Start Universal SYBR Green Master (ROX) kit (Roche Diagnostics, Mannheim, Germany) and the Applied Biosystems 7500 Real-Time PCR System (Applied Biosystems, Foster City, Calif., USA). Relative expression levels were calculated by using the ΔΔC_T_ method after normalization to the housekeeping gene *GAPDH* (*D-glyceraldehyde-3-phosphate dehydrogenase*).

Semi-quantitative RT-PCR analysis was performed with TaKaRa ExTaq (TaKaRa Bio, Shiga, Japan) for 30 cycles, with each cycle consisting in 94°C for 30 s, 65°C for 30 s and 72°C for 20 s, followed by an additional 10 min incubation at 72°C after completion of the last cycle. The transcript level of the *dystrophin* gene was standardized to the ribosomal RNA *18S* gene level. The primer sequences used for qPCR and RT-PCR are listed in Supplementary Table 1. For each experiment, at least two independent qPCR and RT-PCR experiments were performed.

### In vitro myogenesis

The method used herein for in vitro myogenesis was modified as previously described (Kawamichi et al. 2010). FTCVs obtained between the second and fifth passages of culture were seeded at a density of 3×10^4^ cells/ml in growth medium (DMEM supplemented with 20% FBS) onto 60-mm collagen-I-coated cell culture dishes (Asahi Glass, Tokyo, Japan). At 48 h after being seeded onto the collagen I-coated dishes, the cells were treated with 5 μM 5-azacytidine (Sigma-Aldrich, St. Louis, Mo., USA) for 24 h. Cell culture was then maintained in differentiation medium (DMEM supplemented with 2% horse serum) for 21 days. The differentiation medium was changed twice a week until the experiment was terminated.

### Dystrophin and myosin heavy chain expression by immunocytochemistry

The FTCVs obtained before 5-azacytidine treatment and 21 days after treatment were rinsed twice with PBS, fixed with 4% paraformaldehyde in PBS at room temperature for 20 min, permeabilized with cold 100% methanol on ice for 10 min and subsequently rinsed with PBS (3×5 min). The cells were treated with PBS containing 10% horse serum at room temperature for 45 min and were then incubated with rabbit polyclonal anti-dystrophin antibody (Abcam, Cambridge, UK) diluted 1:100 with mouse monoclonal anti-sarcomeric myosin heavy chain protein (MF20; Developmental Studies Hybridoma Bank, University of Iowa) overnight at 4°C. The cells were then washed three times with PBS for 10 min and incubated with secondary antibodies (goat anti-rabbit IgG [H + L] Alexa Fluor 488 and goat anti-mouse IgG [H + L] Alexa Fluor 594 conjugate; Molecular Probes, Eugene, Ore., USA), which were diluted 1:400 with PBS containing 2% bovine serum albumin, for 30 min at room temperature. After this incubation, the cells were washed with PBS three times, treated with Hoechst dye (0.1 μg/ml) at room temperature for 10 min, washed with PBS once, observed under a fluorescence microscope (ECLIPSE TE2000-U; Nikon, Tokyo, Japan) and then photographed by using an AxioCam HRC (Carl Zeiss MicroImaging, Tokyo, Japan). All micrographs were taken under identical conditions with the same exposure time. Three independent experiments were performed in triplicate. The fusion index was determined by calculating the percentage of nuclei in MF20-positive (MF20+) myotubes in five randomly encountered fields (a magnification of ×200 per field) per well in triplicate wells.

### Pluripotent marker expression by immunocytochemistry

The FTCVs obtained before 5-azacytidine treatment and 1 day after treatment were rinsed twice with PBS, fixed with 4% paraformaldehyde in PBS at room temperature for 20 min and permeabilized with 0.1% Triton X-100 in PBS for 10 min and subsequently rinsed with PBS (3×5 min). The cells were treated with PBS containing 10% goat serum at room temperature for 45 min and were then incubated with primary antibodies (rabbit monoclonal anti-Nanog, Oct4, Sox2 antibody; Cell Signaling Technology, Danvers, Mass., USA) diluted 1:400 with 10% goat serum in PBS overnight at 4°C. Then, the cells were washed three times with PBS for 10 min and incubated with secondary antibodies (goat anti-rabbit IgG [H + L] Alexa Fluor 488 and goat anti-mouse IgG [H + L] Alexa Fluor 594 conjugate; Molecular Probes), which were diluted 1:400 with PBS containing 2% bovine serum albumin, for 30 min at room temperature. After this incubation, the cells were washed with PBS three times, treated with Hoechst dye at room temperature for 10 min, washed with PBS once and then observed under a fluorescence microscope.

### Western blotting

The FTCVs obtained before 5-azacytidine treatment and 21 days after treatment on 60-mm collagen-I-coated dishes were scraped in 1 ml homogenizing buffer consisting in 0.02 mol/l NaOH, 1% sodium dodecyl sulfate (SDS, Sigma-Aldrich), 5 mM O,O′-Bis [2-aminoethyl] ethyleneglycol-N, N, N′, N′-tetraacetic acid (Dojindo Molecular Technologies, Kumamoto, Japan) and Complete Mini (Roche Diagnostics), on ice and the cells were collected. Homogenates were collected in separate microcentrifuge tubes and centrifuged at 15,000*g* for 30 min at 4°C. Equal amounts of protein (10 μg) were loaded onto SDS-polyacrylamide gels and blotted onto Immobilon-P membranes (Millipore, Bedford, Mass., USA) by using a semi-dry transfer system (Atto, Tokyo, Japan). The blots were incubated with primary antibodies against dystrophin (NCL-DYS2 [1:200] and NCL-DYS3 [1:50; Novocastra Laboratories, Newcastle upon Tyne, UK]) for 3 h at room temperature and were subsequently washed three times with blocking buffer. The blots were then incubated with a peroxidase-conjugated goat anti-mouse IgG (H + L) secondary antibody (0.05 μl/ml; Jackson ImmunoResearch Laboratories, West Grove, Pa., USA; directed against the primary antibody) for 2 h at room temperature. The membranes were subsequently treated with an enzyme chemiluminescence assay (ECL plus Western Blotting Detection System; GE Healthcare, Buckinghamshire, UK) and the reactions were visualized by exposing the membranes to an X-ray film (Hyperfilm ECL; GE Healthcare) overnight.

## Results

### Expression of mesenchymal stem cell markers in undifferentiated FTCVs and TTCVs

**Fig. 1:**
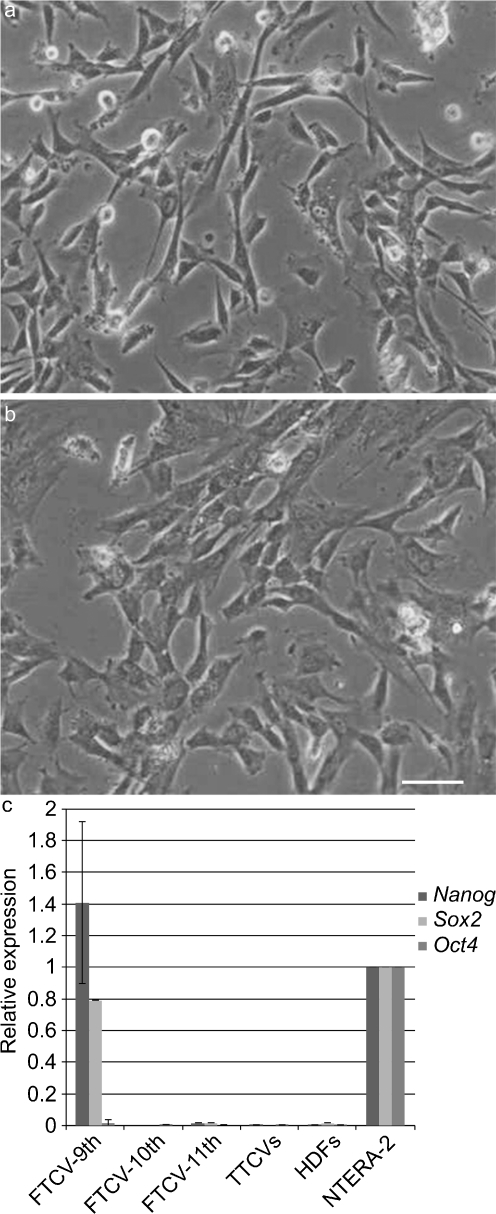
Phase contrast microscopic images of undifferentiated first-trimester chorionic-villi-derived cells (FTCVs; **a**) and third-trimester placental chorionic-villi-derived cells (TTCVs; **b**). *Bar* 50 μm. **c** Quantitative real-time reverse transcription with polymerase chain reaction (qPCR) analysis for *Nanog*, *Sox2* and *Oct4* mRNA expression in FTCVs at the 9th (FTCV-9th), 10th (FTCV-10th) and 11th (FTCV-11th) weeks of gestation, TTCVs, normal human dermal fibroblasts (HDFs) and a pluripotent human embryonic carcinoma cell line (NTERA-2). The value for NTERA-2 was set to 1 in each experiment. Each value (*n*=4) represents the mean ± SD

We successfully cultured a large number of primary cells from FTCVs and TTCVs and found that these cells showed a fibroblast-like morphology at the second passage of culture (Fig. 1a, b). After isolation of the cells, we immunocytochemically evaluated the expression of the surface markers present on the uninduced FTCVs and TTCVs from three individuals by flow cytometry (Table 1). Both FTCVs and TTCVs were positive for MSC markers CD29, CD44, CD73 and CD105 but were negative for the CD34 hematopoietic marker, indicating that both the FTCV- and TTCV-cultured populations were depleted of hematopoietic cells. Furthermore, we found that the average level of MSC marker expression on the FTCVs was higher than that on the TTCVs but this difference was not statistically significant.

**Table 1:** Flow cytometric analysis of primary cultures without inductive stimuli. Percentages of TTCVs and FTCVs immunostained for CD29, CD44, CD73, CD105 and CD34 (mean ± SD, *n*=3; *FITC* fluorescein isothiocyanate, *PE* phycoerythrin, *PC5* phycoerythrin-cyanine 5)

Cells	CD29 FITC	CD44 FITC	CD73 PE	CD105 PE	CD34 PC5
TTCVs	96.9±2.2	94.7±4.86	87.3±10.8	91.5±9.25	0.27
FTCVs	98.8±1.0	97.2±1.89	93.6±4.23	98.1±2.03	0.27

### Expression of pluripotent stem cell markers by FTCVs

**Fig. 2:**
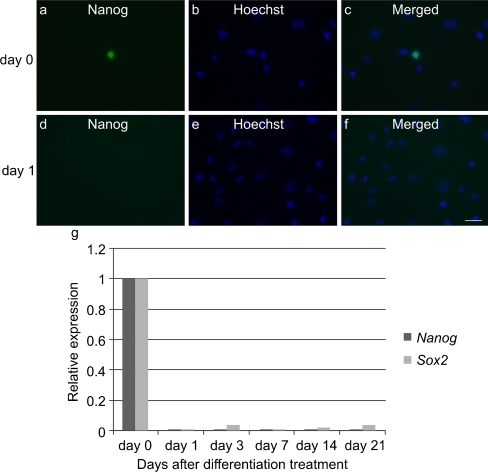
Immunocytochemistry of the Nanog protein. **a** Nanog (day 0). **b** Hoechst dye (day 0). **c** Merged image of **a**, **b** (day 0). **d** Nanog (day 1). **e** Hoechst dye (day 1). **f** Merged image of **d**, **e** (day 1). *Bar* 50 μm. **g** qPCR analysis of *Nanog* and *Sox2* expression in FTCV-9th cells. Time course of *Nanog* and *Sox2* mRNA expression at the indicated day after treatment with 5-azacytidine. The value obtained before treatment with 5-azacytidine was set to 1 in each experiment

We also analyzed the gene expression of pluripotent stem cell markers in uninduced FTCVs and TTCVs obtained between the 3rd and 6th passages of culture by qPCR analysis. FTCVs collected during the 9th week of gestation (FTCV-9th) expressed both *Nanog* and *Sox2* mRNA but not *Oct4* mRNA (Fig. 1c). In contrast, FTCVs collected during later weeks of gestation (FTCV-10th, FTCV-11th) and TTCVs exhibited no detectable expression of these pluripotent stem cell markers. Moreover, immunocytochemical analysis revealed the presence of the Nanog protein in uninduced FTCV-9th cells (Fig. 2a).

### Myogenic differentiation

**Fig. 3:**
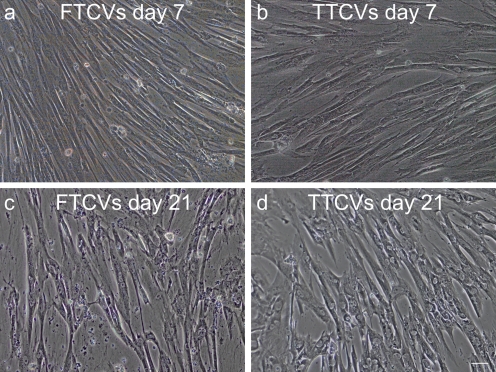
Phase contrast microscopic images of the FTCVs and TTCVs after treatment with 5-azacytidine. **a** FTCVs (day 7). **b** TTCVs (day 7), **c** FTCVs (day 21), **d** TTCVs (day 21). *Bar* 50 μm

**Fig. 4:**
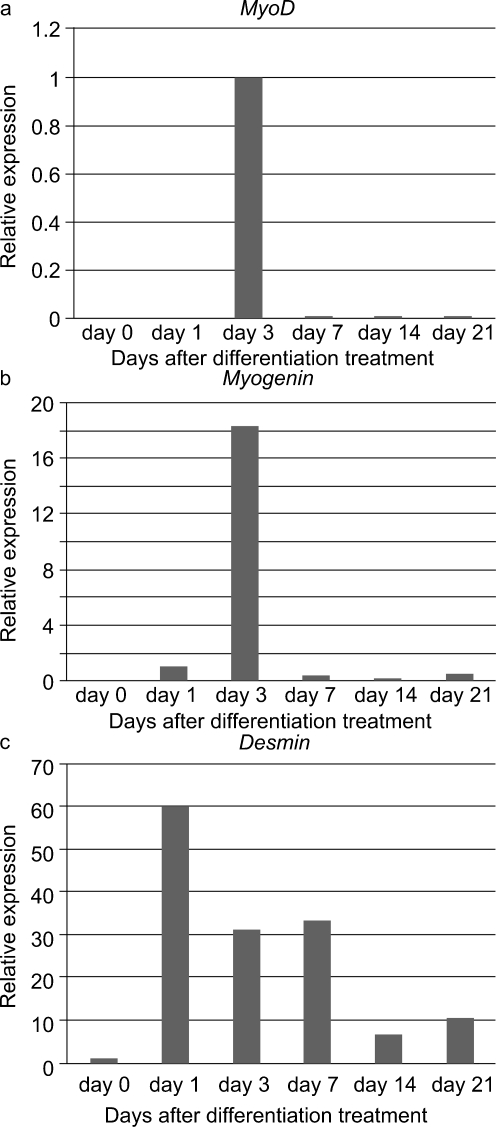
qPCR analysis of skeletal muscle markers in the FTCVs. Time course of *MyoD* (**a**), *myogenin* (**b**) and *desmin* (**c**) mRNA expression at the indicated day after treatment with 5-azacytidine. *MyoD* mRNA was detectable after day 3. *Myogenin* mRNA was undetectable at day 0. The earliest detectable value was set to 1 in each experiment

**Fig. 5:**
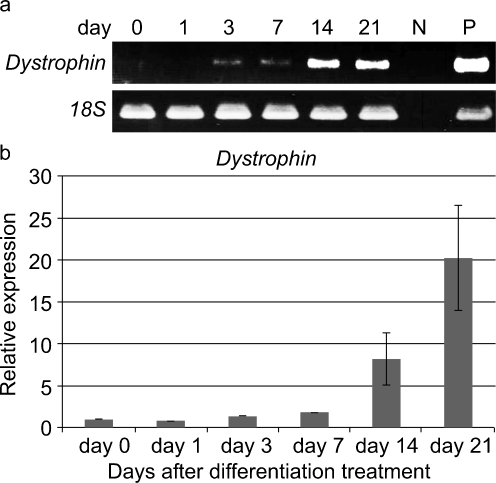
Expression of *dystrophin* mRNA in the FTCVs during myogenic induction. **a** RT-PCR analysis. Time course of *dystrophin* and ribosomal RNA *18S* mRNA expression at the indicated day after treatment with 5-azacytidine (*N* negative control lanes without reverse transcriptase, *P* RNAs from human skeletal muscle as positive control). **b** qPCR analysis. Time course of *dystrophin* mRNA expression at the indicated day after treatment with 5-azacytidine. The value obtained before treatment with 5-azacytidine was set to 1. Each value (*n*=3) represents the mean ± SD

**Fig. 6:**
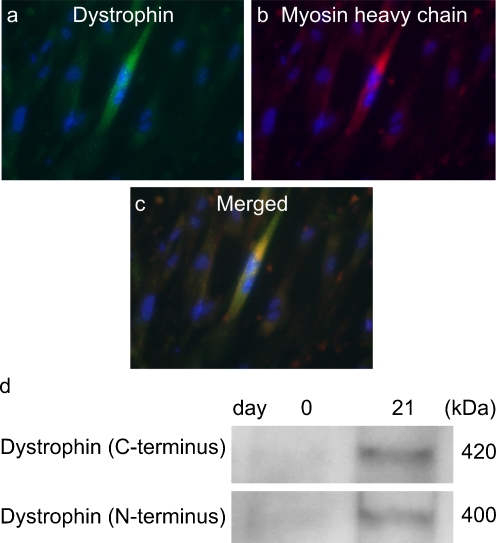
Immunocytochemistry of dystrophin (*green*) and myosin heavy chain (*red*) proteins in FTCVs at 21 days after 5-azacytidine treatment. The nuclei were counterstained with Hoechst dye (*blue*). **a** Dystrophin. **b** Myosin heavy chain (MF20). **c** Merged image of **a**, **b**. *Bar* 50 μm. **d** Western blot of dystrophin (using C-terminus [NCL-DYS2] and N- terminus [NCL-DYS3] antibodies) in the FTCV lysate before 5-azacytidine treatment (day 0) and at day 21 after 5-azacytidine treatment

**Fig. 7:**
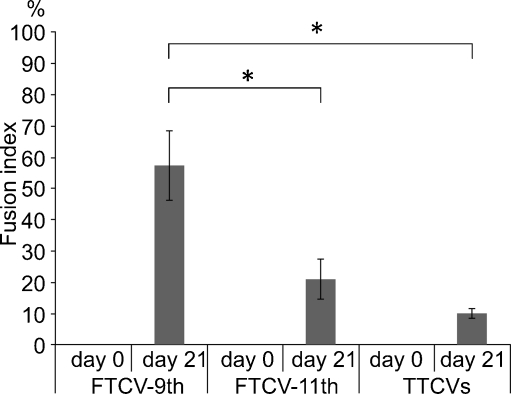
Quantification of MF20-positive (MF20+) cells in FTCV-9th cells, FTCV-11th cells and TTCVs during in vitro differentiation. Fusion index was determined by calculating the percentage of nuclei among MF20+ cells, as described. Each data point (*n*=3) is presented as the mean ± SD. Quantification of MF20+ cells in each population was compared by using the Student’s *t*-test (*, *P* < 0.05)

To evaluate their myogenic potential, the differentiation of the FTCVs into a myogenic lineage was induced by incubation with 5-azacytidine in DMEM with 20% FBS for 24 h, after which they were cultured in DMEM supplemented with 2% horse serum for 21 days. We found that FTCV expression of both *Nanog* and *Sox2* mRNA decreased markedly after 1 day of myogenic induction (Fig. 2g); indeed, the Nanog protein was also down-regulated after myogenic induction (Fig. 2d). During myogenic induction, the morphology of the FTCV population changed to multinucleated myotubes (Fig. 3). We evaluated the expression of the myogenic markers *MyoD*, *myogenin*, *desmin* and *dystrophin* in the FTCVs by qPCR analysis. These experiments revealed that myogenic differentiation of the FTCVs induced both *MyoD* and *myogenin* mRNA expression by day 3, although this expression decreased again by day 7. Moreover, FTCV expression of *desmin* mRNA was relatively high on day 1 of myogenic differentiation and subsequently decreased to a moderate level (Fig. 4). In addition, RT-PCR analysis clearly showed that *dystrophin* mRNA expression was increased from day 14 (Fig. 5a), as confirmed by qPCR analysis (Fig. 5b). Immunocytochemistry and Western blot analysis also confirmed that the dystrophin protein was detected in the FTCVs at day 21 (Fig. 6a, d). To evaluate the efficiency of myotube formation, FTCVs and TTCVs were fixed at day 0 and day 21 after plating and stained as described in Materials and methods with an antibody to the myosin heavy chain protein (MF20) in order to determine the fusion index. The results revealed that FTCV-9th cells with a fusion index of 57.3±11.1% had significantly higher myogenicity than FTCV-11th cells (20.9±6.4%) and TTCVs (9.9±1.4%) at day 21 (Fig. 7).

### In vitro differentiation of multiple lineages

**Fig. 8:**
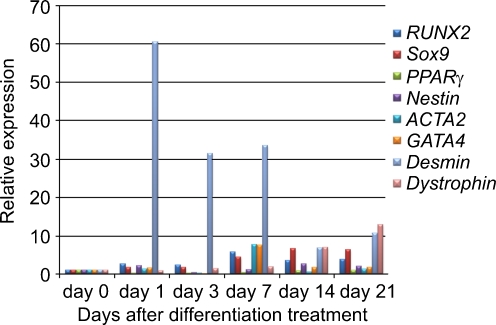
qPCR analysis of multiple tissue markers in FTCVs. Time course of *RUNX2*, *Sox9*, *PPARγ*, *nestin*, *ACTA2* and *GATA4* and skeletal muscle marker (*desmin* and *dystrophin*) mRNA expression at the indicated day after treatment with 5-azacytidine. The value obtained before treatment with 5-azacytidine was set to 1 in each experiment

qPCR analysis revealed that FTCVs treated with 5-azacytidine and maintained in myogenic culture conditions also expressed *RUNX2*, *Sox9*, *PPARγ*, *nestin*, *ACTA2* and *GATA4*, which are markers of the osteoblast, chondrocyte, adipocyte, neural, smooth muscle and cardiac lineages, respectively (Fig. 8). Furthermore, the relative levels of expression of these non-myogenic markers were lower than the expression levels of myogenic markers such as *desmin* and *dystrophin*.

## Discussion

In this study, we have investigated the myogenic potential of FTCVs as a cell source for muscular dystrophy cell therapy. To our knowledge, no previous studies have examined the effect of combining 5-azacytidine treatment with culture in myogenic differentiation media on the expression of dystrophin in FTCVs. Recently, stem cells have been investigated intensively for their potential use in various therapeutic regimens, including tissue engineering and cell therapy. Stem cells can be loosely classified into three groups: embryonic stem (ES) cells, fetal stem cells (i.e., first-trimester chorionic villi and placenta) and adult stem cells. In particular, FTCVs possess a number of advantages that contribute to their potential therapeutic use. First, FTCVs might have a higher myogenic potential for differentiation than cells taken from other tissues because they are derived from the earliest fetal cell population. Second, the ability to isolate pluripotent autogenic progenitor cells such as FTCVs during gestation might be advantageous for the timely treatment of genetic diseases in newborns. Third, fetal cells do not form teratomas when injected into adults and are less immunogenic than other cell population, making them particularly suitable for transplantation (In’t Anker et al. 2003; De Coppi et al. 2007). Fourth, the use of FTCVs for treatment avoids the ethical issues associated with ES cells (Weiss and Troyer 2006). Finally, FTCVs are obtained within the process of prenatal diagnosis and can be taken without an additional invasive procedure.

Our present results suggest that FTCVs express *Nanog* and *Sox2* mRNA, the proteins of which have been previously identified as crucial factors for maintaining stem cell characteristics in human ES cells (Hoffman and Carpenter 2005; Kazuki et al. 2010). The expression level of *Nanog* mRNA in the FTCVs collected during the 9th week of gestation is not significantly different from that of NTERA-2 cells, which express pluripotent stem cell markers (Pal and Ravindran 2006; Fig. 1c). Our investigation of pluripotent stem cell marker expression in the FTCVs collected between the 9th and 11th weeks of gestation has revealed that both *Nanog* and *Sox2* mRNA are expressed in the cells collected during the 9th week. Immunocytochemistry has revealed that Nanog-protein-positive cells constitute only a small population of the FTCV-9th cells (Fig. 2a). Because FTCVs exhibit heterogeneity, which suggests the presence of other stem-cell-like cells, additional studies are required to assess further the expression pattern of stem cell markers. However, these data confirm that the FTCV-9th cells contain a subpopulation of uncommitted progenitors that express *Nanog* and *Sox2* mRNA and that they are likely to be distinct from the committed MSC population. We have also confirmed that FTCVs expressed MSC markers at a high level (Table 1). Although the FTCVs from the 10th and 11th weeks do not express *Nanog* and *Sox2* mRNA, they do express myogenic markers, including *dystrophin*, after myogenic induction. These data indicate that FTCVs possibly down-regulate the expression of *Nanog* and *Sox2* mRNA between the 9th and 10th weeks of gestation (Fig. 1c). On the basis of these findings, we conclude that the characteristics of our FTCV-9th population require further detailed analysis. Furthermore, our results indicate that FTCVs can be efficiently directed to differentiate in vitro into skeletal muscle cells that express dystrophin (Fig. 6). Dystrophin is a mature skeletal muscle protein produced during cell fusion (Edmondson and Olson 1993).

Under the myogenic differentiation conditions used in this study, FTCVs exhibit a greater increase in the expression of myogenic markers than any other lineage markers (Fig. 8). Indeed, MSCs derived from TTCVs can be induced to differentiate into skeletal muscle by treatment with 5-azacytidine in DMEM with 20% FBS and subsequent culture in 2% horse serum. Initially, we optimized the cell concentration used in the present study from this previously reported culture method (Kawamichi et al. 2010) but our modified method led to the further formation of myotubes in the FTCVs. Although such myogenic induction was probably caused by transient and random gene modifications attributable to treatment with the DNA demethylation agent 5-azacytidine, our myogenic culture conditions might have increased the effectiveness of myogenesis in the FTCVs.

Since DMD-derived FTCVs are regularly obtained during early pregnancy for prenatal diagnosis purposes, they are a stable source of cells with pluripotent and myogenic differentiation potential. Furthermore, the DMD-derived FTCVs can be easily cultured in vitro. These cells can be manipulated by using gene-targeting protocols that involve a mini-dystrophin gene (Tang et al. 2010), other genes using viral vectors (Odom et al. 2008), or antisense morpholinos (Saito et al. 2010). Alternatively, they can be treated with pharmacological agents, such as aminoglycoside (Barton-Davis et al. 1999; Malik et al. 2010) or negamycin (Arakawa et al. 2003; Allamand et al. 2008), which target the nonsense mutation in the *dystrophin* gene of about 15% of DMD patients. These particular properties of the FTCVs might enable effective DMD treatment by allowing the use of a patient’s own FTCVs for self-transplantation with one of the above-mentioned gene therapies. Myogenic differentiation of reimplanted FTCVs *in situ* could theoretically repopulate and repair the dysfunctional skeletal muscles in DMD. Furthermore, a combination of targeted gene manipulation and pharmacological treatments that enable stem cells to grow and differentiate indefinitely could further extend the possible therapeutic potential of these cells. Moreover, such manipulations of stem cells offer the potential of developing protocols to cure genetic defects and to assess in great detail the functionality and safety of therapeutic protocols in vitro. Indeed, patient-derived FTCVs might be suitable for self-transplantation with nonimmunogenicity or low immunogenicity and non-tumorigenicity. Even FTCVs with a different genetic background might represent a novel cell source for transplantation because of their low immunogenicity and lack of need for immunosuppressive agents.

The expression of markers for MSCs, pluripotent stem cells and myogenic cells in the FTCV population opens up new possibilities in cell therapy for DMD patients and individuals with other neuromuscular diseases. Further in vivo studies, which are currently under way, will be essential for identifying the factors that determine the conditions for definitive myogenic differentiation, survival and dissemination and the potential clinical effects of these cells in animal models.

In summary, FTCVs that have been previously obtained for prenatal diagnosis express markers of MSCs and pluripotent stem cells and undergo myogenesis efficiently in vitro. The accessibility of FTCVs and the minimal ethical issues involved with obtaining these cells make them promising therapeutic candidates for patients suffering from DMD.

## Electronic supplementary material

Below is the link to the electronic supplementary material.

Supplementary Table 1 Primer sequences for quantitative real-time RT-PCR and semiquantitativeRT-PCR (DOC 85 kb)
