# Supplementary material for: Human first-trimester chorionic villi have a myogenic potential
Source: Cell Tissue Res. 2012 Feb 28;348(1):189–97. doi: 10.1007/s00441-012-1340-9 (PMC3316778; doi:10.1007/s00441-012-1340-9)
Supplement: Supplementary file 1 — Primer sequences for quantitative real-time RT-PCR and semiquantitativeRT-PCR (DOC 85 kb) [file 441_2012_1340_MOESM1_ESM.doc]

**Supplementary Table 1. Primer sequences for quantitative real-time RT-PCR and semi-quantitative RT-PCR**

| Gene |  | Sequence |
| --- | --- | --- |
| *Oct4* | Forward | GACAGGGGGAGGGGAGGAGCTAGG |
|  | Reverse | CTTCCCTCCAACCAGTTGCCCCAAAC |
| *Sox2* | Forward | GGGAAATGGGAGGGGTGCAAAAGAGG |
|  | Reverse | TTGCGTGAGTGTGGATGGGATTGGTG |
| *Nanog* | Forward | TCTCTCCTCTTCCTTCCTCCATG |
|  | Reverse | CTGTTTGTAGCTGAGGTTCAGGATG |
| *MyoD* | Forward | GCAGGTGTAACCGTAACC |
|  | Reverse | ACGTACAAATTCCCTGTAGC |
| *Myogenin* | Forward | GCCACAGATGCCACTACTTC |
|  | Reverse | CAACTTCAGCACAGGAGACC |
| *Desmin* | Forward | CCTACTCTGCCCTCAACTTC |
|  | Reverse | AGTATCCCAACACCCTGCTC |
| *Dystrophin* | Forward | GAGGCTGTAAGGAGGCAAAAGTTG |
|  | Reverse | TCAAGTTCTTTGGGATTTTCCGTC |
| *18S* | Forward | GTGGAGGGATTTGTCTGGTT |
|  | Reverse | CGCTGAGCCAGTCAGTGTAG |
| *Runt-related transcription factor 2* | Forward | AACCCTTAATTTGCACTGGGTCA |
| *(RUNX2)* | Reverse | CAAATTCCAGCAATGTTTGTGCTAC |
| *Sox9* | Forward | GGAGATGAAATCTGTTCTGGGAATG |
|  | Reverse | TGAAGGTTAACTGCTGGTGTTCTGA |
| *Peroxisome proliferator activated receptor γ* | Forward | TGGAATTAGATGACAGCGACTTGG |
| *(PPARγ )* | Reverse | CTGGAGCAGCTTGGCAAACA |
| *Nestin* | Forward | CTCCAAGAATGGAGGCTGTAGGAA |
|  | Reverse | CCTATGAGATGGAGCAGGCAAGA |
| *α-smooth muscle actin* | Forward | ATTGCCGACCGAATGCAGA |
| *(ACTA2)* | Reverse | ATGGAGCCACCGATCCAGAC |
| *GATA binding protein 4* | Forward | CGGAAGCCCAAGAACCTGAATA |
| *(GATA4)* | Reverse | GCTGGAGTTGCTGGAAGCAC |
| *GAPDH* | Forward | GCACCGTCAAGGCTGAGAAC |
|  | Reverse | TGGTGAAGACGCCAGTGGA |
